# A Rare Case of Pyriform Sinus Fistula in an Adult

**DOI:** 10.7759/cureus.25654

**Published:** 2022-06-04

**Authors:** Hawwa Reesha, Prakash Adhikari, Jennifer L Madeo

**Affiliations:** 1 Internal Medicine, Piedmont Athens Regional Medical Center, Athens, USA; 2 Infectious Diseases, Piedmont Athens Regional Medical Center, Athens, USA

**Keywords:** abscess, neck, retropharyngeal abscess, fistula, pyriform sinus

## Abstract

The pyriform sinus fistula is very rare in adults, and it can present as recurrent deep neck space infection and abscess formation. The fistula results due to failure of obliteration of the third and fourth pharyngeal pouches. The diagnosis is often challenging, even with standard imaging techniques. We present a case of a patient who developed recurrent neck space infection and abscess formation. She was found to have a pyriform sinus fistula with fluoroscopy esophagram, although it was not seen with a standard contrast-enhanced computed tomography scan of the neck.

## Introduction

The pyriform sinus fistula is an epithelialized tract connecting the skin of the neck to the foregut [1]. This is a rare congenital condition due to disturbances in the development of the fetal branchial apparatus, specifically, failure to obliterate the third and fourth pharyngeal pouches. Interestingly an external opening to the skin is rarely present [2]. The pyriform sinus fistula is prone to recurrent deep neck space infection and abscess formation [3]. We present a case of an adult who had recurrent neck space infection and abscess formation secondary to a pyriform sinus fistula. This case is of interest due to its rarity and difficulty in diagnosis. It should be considered in the differential diagnosis in patients with recurrent neck infections.

## Case presentation

A 40-year-old female presented to the emergency department with neck pain and swelling for three days. Her symptoms began with irritation at the back of the throat followed by some odynophagia to solid foods. She complained of chills but denied fever or shortness of breath. There was no history of neck trauma or sick contact. She was a former smoker, quit smoking cigarettes six years ago, and drinks alcohol only on social occasions. She has no chronic medical condition, and she does not take over-the-counter or prescription medications. She was admitted to the hospital two months ago with a similar presentation. At that time, she was diagnosed with a neck abscess based on imaging findings. Incision and drainage of the abscess were done and then she completed a 14-day course of antibiotics. Upon further exploration of her history, she had three more episodes of neck abscess in the past where she was treated with a one to two weeks course of antibiotics only.

On examination, her vital signs were normal, and there was an area of tenderness in the anterior neck but there was no visible erythema or draining sinus. The oral cavity examination was unrevealing. Complete blood count (CBC) was significant for white blood cell (WBC) count of 13,200/µL (normal 4000-11000/µL) and lactic acid was normal. Contrast-enhanced computed tomography (CT) of the neck showed a left-sided neck abscess with associated cellulitis, myositis of sternocleidomastoid, and strap musculature. Flexible fiberoptic laryngoscopy demonstrated mucus secretions from the left piriform sinus; however, no pits were seen suggestive of the branchial cleft sinus. Given her recurrent similar episodes, pyriform sinus fistula was high on our differential diagnosis.

Vancomycin and tazobactam-piperacillin were initially administered. A short course of intravenous dexamethasone was also given to combat neck edema and swelling. She then underwent incision and drainage. Laryngoscopy at the time of surgery also did not show a fistula. A surgical sample was sent for culture and *Peptostreptococcus*, *Actinomyces*, and *Prevotella* species were identified; all organisms commonly implicated in deep neck space infection. Blood cultures were negative. Antibiotics were changed to ampicillin-sulbactam based on the susceptibility results. The swelling of the neck gradually subsided and she improved within the next two days.

**Figure 1:**
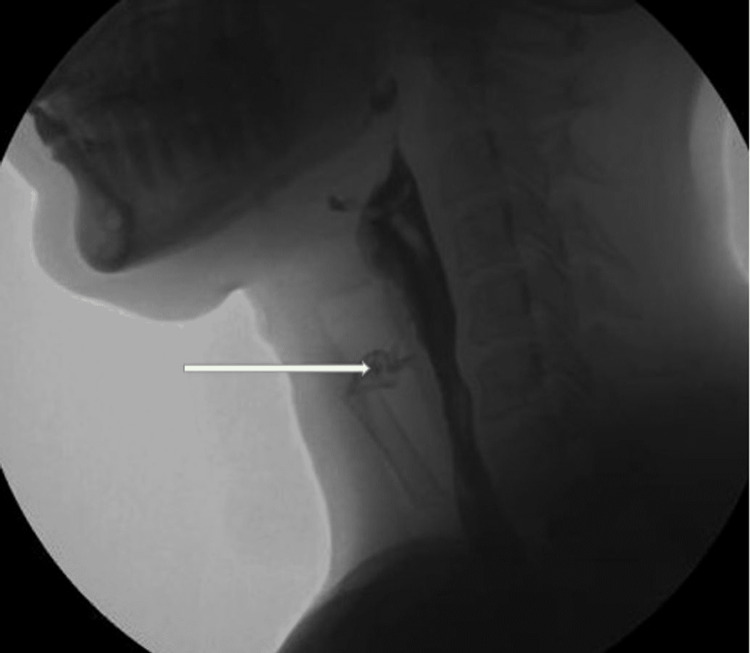
Fluoroscopy esophagogram. White arrow showing thin leak of contrast material from the inferior apex of the pyriform sinus to the left of the esophagus to the medial aspect of the indwelling Penrose drain.

Due to the recurrent nature of the neck abscess in an otherwise healthy female, additional evaluation was conducted. A fluoroscopy esophagram was performed and showed a thin leak of contrast material from the inferior apex of the pyriform sinus to the left of the esophagus, confirming the presence of pyriform sinus fistula (Figure 1). She was then referred to an otolaryngologist who specialized in pyriform sinus fistula treatment. Two weeks after completing the course of antibiotics, she underwent endoscopic cauterization as definitive management.

## Discussion

Deep neck space infection with abscess formation can be a potentially life-threatening condition and can easily spread to the superior mediastinum via the parapharyngeal space. Deep neck space abscess is more common in children under the age of five, who have an antecedent upper respiratory infection [4]. Trauma to the posterior pharynx, poor oral hygiene, diabetes, and immunocompromised state are some causes of neck abscess in older children and adults. Rarely, contiguous spread from osteomyelitis and spinal discitis can cause retropharyngeal abscess [4].

Pyriform sinus fistula is an extremely rare cause of recurrent deep neck space infection and abscess formation. It results from the persistence of the pharyngobranchial duct that connects the third and fourth pharyngeal pouches. Normally, the pyriform sinus degenerates in the seventh week of embryonic development [2]. Pyriform sinus fistula presents as latero-cervical swelling, especially on the left side, dysphagia, sore throat, hoarseness, and upper respiratory infection [5]. This fistula serves as a pathway for the spread of infection, leading to recurrent abscess formation, cellulitis, and even mediastinitis [6]. Acute suppurative thyroiditis, neck mass with and without respiratory distress, and cutaneous discharging fistula with and without infection are some other presenting symptoms [7]. Treatment with surgeries, such as incisions and drainage, can lead to the development of external sinus openings [8].

Diagnosis is often challenging and requires a high index of suspicion. Imaging modalities including CT, magnetic resonance imaging (MRI), ultrasound, and barium swallow are utilized in securing a diagnosis. Contrast-enhanced CT is known to be superior in detecting air density along the fistulous tract. Similarly, barium esophagography (sensitivity: 50%) can be used for the evaluation of sinus tracts [9]. Our patient showed typical clinical features with recurrent left-sided neck abscesses, first starting eight years ago. The contrast-enhanced CT of the neck demonstrated findings of abscess and cellulitis but failed to identify the fistula. We were able to identify the fistula with a fluoroscopy esophagram.

The definitive treatment of pyriform sinus fistula with recurrent infection is surgical excision [6]. The excision is usually performed several weeks after the resolution of acute infection. Less invasive methods like chemo-cauterization or electrocauterization can also be used but these methods have a higher rate of treatment failure [4].

## Conclusions

The pyriform sinus fistula is a rare condition, but clinical suspicion should be high in patients with a recurrent history of deep neck infection and abscess formation. Diagnosis is often challenging, contrast-enhanced CT scan or MRI is the study of choice. Fluoroscopy esophagram can be performed to demonstrate the fistula. Surgery is the most effective treatment modality to prevent recurrent neck infections.
